# Training Colonies

**Published:** 1920-02-07

**Authors:** 


THE PUBLIC WELL-BEING—(*continued*).

### TRAINING COLONIES.

### Plans for Consumptives and Ex-Service Men.

The problem of securing the utmost possible permanence of good results following sanatorium treatment for tuberculosis has exercised many minds during the past few years, and the annual report of the Medical Department of the Local Government Board says a strong feeling has grown in favour of the arrangements for graduated labour at sanatoria being developed on training colony lines, with the idea of providing, where necessary, new occupations to be carried on under open-air conditions by the phthisical patient on leaving the institution. Until recently undue stress has been laid on training in agricultural work. It is now generally recognised that, except for a very limited number of cases, general agricultural work is too laborious for the ex-consumptive, and that he can only be put to the lighter forms of farm labour. The farm colony idea has, therefore, developed into the wider conception of the training or occupational colony.

A commencement has also been made with schemes for providing permanent village settlements in which tuberculosis patients, after a period of treatment in a sanatorium and training in a colony, can carry out their new employment under suitable conditions. Thus, sanatorium treatment should merge into occupational training, and occupational training result in permanent employment at a suitable occupation under satisfactory conditions, *e.g.*, in a village settlement.

A report by Dr. J. E. Chapman on training colonies was issued during the year. In this report Dr. Chapman pointed out that colony treatment is a natural development of sanatorium treatment to secure for selected patients more permanently beneficial results than can be obtained by ordinary sanatorium life alone. He indicated that at present colonies usually deal with the more hopeful cases only. They afford prolonged treatment, under simplified sanatorium conditions, and provide the inmates with suitable employment, or training in new occupations at which they can earn a living after discharge. Under existing circumstances only patients without dependants or ex-Service men with pensions are usually in a position to take advantage of colony treatment. The utility of training colonies will continue to be limited to these classes unless arrangements can be made for the maintenance of dependants during the residence of bread-winners in the colonies.

He further suggested that suitably situated colonies might be used with advantage (*a*) as halfway houses between the sanatorium and ordinary life for patients who are liable to early relapse, and (*b*) for the accommodation of irrecoverable patients whose working capacity is temporarily or permanently impaired. In the latter case the provision of suitable part-time work and of suitable housing for the patients and their families are needed. He referred to the fact that experience in this form of treatment is somewhat limited, and recommended that local schemes should be begun on a relatively small scale and be developed in the light of experience. The report included a section on the housing of tuberculous patients, accompanied by plans of cottages so designed as to be particularly suitable for families containing one or more tuberculous members, though not containing features so unusual as to hinder their use for normal, healthy people or to attract attention unduly.

In this connection we print to-day two schemes—one of them a wise consideration of the problem by the Medical Officer of Health for Derbyshire, and the other the definite scheme for the special care of tuberculous sailors and soldiers, definitely set on foot by the National Society for the Prevention of Consumption.

